# Multiple myeloma presenting as cutaneous leukocytoclastic vasculitis and eosinophilia disclosing a T helper type 1/T helper type 2 imbalance: a case report

**DOI:** 10.1186/s13256-018-1857-y

**Published:** 2018-10-31

**Authors:** Satoko Oka, Kazuo Ono, Masaharu Nohgawa

**Affiliations:** 1 Division of Hematology, Japanese Red Cross Society Wakayama Medical Center, Wakayama, Japan; 2 Division of Pathology, Japanese Red Cross Society Wakayama Medical Center, Wakayama, Japan

**Keywords:** Multiple myeloma (MM), Leukocytoclastic vasculitis (LV), Eosinophilia, Th1/Th2

## Abstract

**Background:**

Multiple myeloma is a very heterogeneous disease comprising a number of genetic entities that differ from each other in their evolution, mode of presentation, response to therapy, and prognosis. Due to its more chronic nature and cumulative toxicities that patients develop from multiple lines of treatments, a number of symptoms are associated with multiple myeloma. However, the mechanisms responsible for the relationship between these symptoms and multiple myeloma currently remain unclear.

**Case presentation:**

An 85-year-old Japanese woman exhibited the rare presentation of multiple myeloma (immunoglobulin kappa chain type) with leukocytoclastic vasculitis and eosinophilia. The serum level of interferon-γ was decreased; however, serum levels of interleukin-4, interleukin-5, interleukin-6, interleukin-10, and tumor growth factor-β levels were elevated. She received a bortezomib, lenalidomide, and dexamethasone regimen. After one course of the treatment, the cutaneous manifestation rapidly improved and laboratory tests showed decrease of eosinophil cell count. Serum concentrations of immunoglobulin G decreased and plasma cells in bone marrow decreased. The serum level of interferon-γ was elevated and serum levels of interleukin-4, interleukin-5, interleukin-6, interleukin-10, and tumor growth factor-β decreased.

**Conclusions:**

It is the first case of leukocytoclastic vasculitis and eosinophilia in multiple myeloma that was associated with a T helper type 1/T helper type 2 imbalance and T regulatory cells, and was successfully treated with bortezomib, lenalidomide, and dexamethasone. The present case reinforces the value of early evaluations for paraneoplastic symptoms in order to reach a diagnosis and allow for the prompt initiation of appropriate treatments and achieve successful therapeutic management.

## Background

The incidence of multiple myeloma (MM) has increased in recent years due to advances in treatments and the overall ageing of society. MM is a very heterogeneous disease comprising a number of genetic entities that differ from each other in their evolution, mode of presentation, response to therapy, and prognosis. Due to its more chronic nature and cumulative toxicities that patients develop from multiple lines of treatments, a number of symptoms are associated with MM. Although the relationship between these symptoms and MM has occasionally been reported in the literature, the underlying mechanisms have not yet been elucidated in detail.

Leukocytoclastic vasculitis (LV) is a systemic inflammatory disorder that mostly involves small vessels. It is characterized by segmental angiocentric neutrophilic inflammation, endothelial cell damage, and fibrinoid necrosis [1]. The development of LV in patients with MM has been linked to cryoglobulinemia, infections, drugs, and paraneoplasia [2–5]; however, the appearance of paraneoplastic vasculitis in the course of MM is rare. Several pathological mechanisms have been proposed to explain the relationship between LV and malignancy, including tumor cells eliciting an immunological reaction against vascular smooth muscle cells that release cytokines, such as interleukin (IL)-6 [6, 7].

Eosinophilia, which may involve peripheral blood or tissues, may be associated with a wide variety of malignant tumors. Among hematopoietic tumors, Hodgkin disease and non-Hodgkin lymphoma of the T cell lineage are the most frequent; however, MM is rarely associated with eosinophilia [8]. T helper type 2 (Th2) cytokines, such as IL-4 and IL-5, are major growth factors for eosinophils.

MM exhibits some properties of immunodeficiency diseases, in which weak immunity is not only related to humoral immune defects mediated by B cells and plasmacytes, but also to cellular immune defects mediated by T cells. The T helper type 1 (Th1)/Th2 balance and T regulatory cells (Treg) play an important role in MM [9, 10] and may be associated with these symptoms.

The present case exhibited the rare presentation of MM with LV and eosinophilia. In addition to a clinicopathological examination, the following serum cytokines were evaluated: interferon (IFN)-γ, IL-4, IL-5, IL-6, IL-10, and tumor growth factor (TGF)-β levels. We describe the first case of LV and eosinophilia in MM that was associated with a Th1/Th2 imbalance and Treg cells, and was successfully treated with bortezomib, lenalidomide, and dexamethasone (VRD).

## Case presentation

In January 2018, an 85-year-old Japanese woman was referred to our hospital with vascular purpura on her lower limbs, chest, and abdomen. She was a housewife and reported no recent travel history. There was no history of urticarial or other allergic symptoms, and she had no familial history. She had no history of smoking tobacco and alcoholism. She had a previous history of hypertension treated with amlodipine besylate for 20 years and no other medication (she was not administered new drugs). She described no trigger factors for purpura. Her vital signs were as follows: temperature, 37.2 °C; pulse, 86 beats per minute; blood pressure, 120/78 mmHg; and respiratory rate, 18 breaths per minute.

**Fig. 1:**
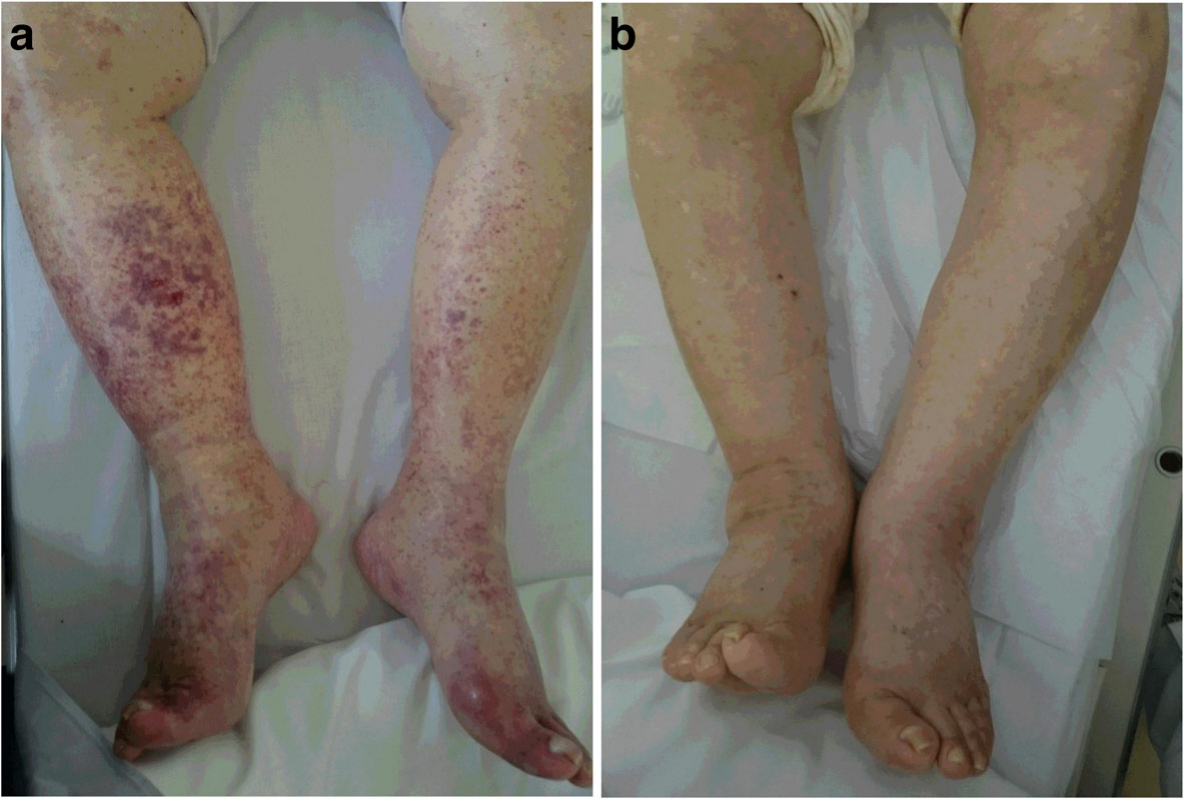
**a** Vascular purpura on the lower limbs. **b** The cutaneous manifestation improved after one course of bortezomib, lenalidomide, and dexamethasone treatment

**Fig. 2:**
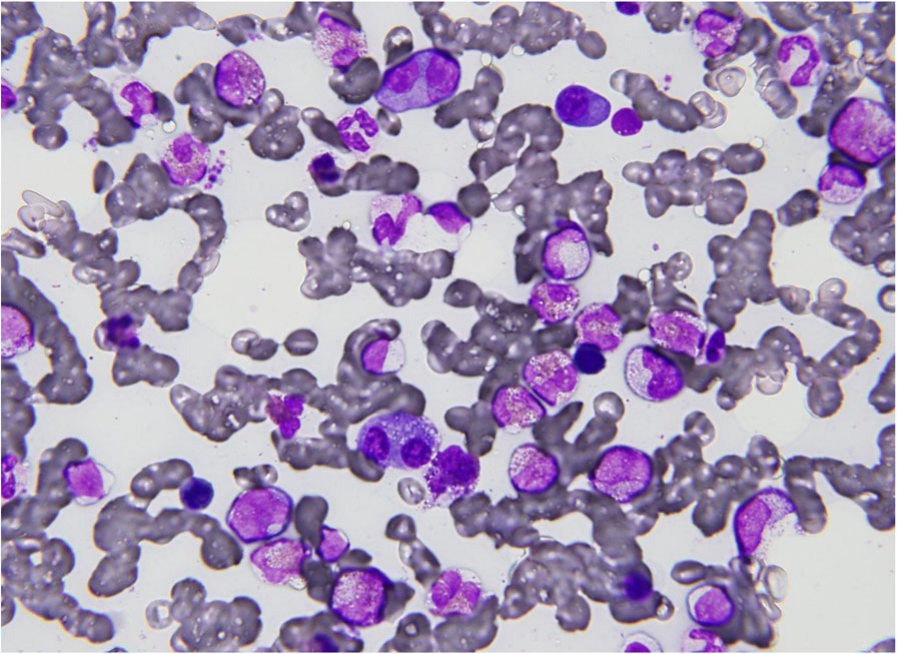
Bone marrow specimen showing increased plasma cells and eosinophils

**Fig. 3:**
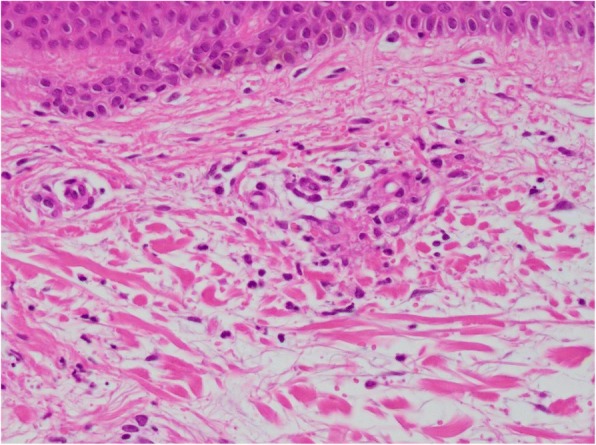
Leukocytoclastic vasculitis showing angiocentric, neutrophilic segmental inflammation with endothelial cell swelling and fibrinoid necrosis on blood vessel walls. A cellular infiltrate around the vessels shows leukocytoclasia of neutrophil nuclei

A physical examination revealed significant pitting edema in both lower legs, and the confluence of palpable purpura that formed several patches of different sizes in her lower limbs, chest, and abdomen (Fig. 1a). There were no remarkable features in her heart, lungs, or abdominal examinations. A neurological examination revealed no abnormalities. She was afebrile and there were no signs of an infectious focus in examinations of each system. Laboratory tests showed a white blood cell count (WBC) of 23.3 × 10^9^/L, eosinophil cell count of 13.5 × 10^9^/L, red blood cell count (RBC) of 299 × 10^10^/L, hemoglobin (Hb) concentration of 9.2 g/dL, and platelet count of 152 × 10^9^/L (Table 1). The serum total protein level was 8.2 g/dL (normal range, 6.9–8.2 g/dL), the lactate dehydrogenase (LDH) level was 280 IU/L (normal range, 106–211 IU/L), the aspartate transaminase level was 50 U/L (normal range, 5–40 U/L), the alanine transaminase level was 40 U/L (normal range, 5–35 U/L), the alkaline phosphatase level was 1564 U/L (normal range, 104–338 U/L), and the C-reactive protein level was 6.6 mg/dL (normal range, below 0.3 mg/dL). The serum creatinine level was 2.1 mg/dL (normal range, 0.40–0.80 mg/dL) and nephrotic-range proteinuria was noted. Hypocomplementemia with elevated C1q levels was observed. Serum antinuclear antibodies, perinuclear anti-neutrophil cytoplasmic antibodies, and cytoplasmic anti-neutrophil cytoplasmic antibodies were all negative. Cryoglobulins and the hepatitis B and C panels were negative. The serum β2-microglobulin level was 13.5 μg/dl (normal range, < 2.0 μg/dl), and immunoglobulin G (IgG), immunoglobulin E (IgE), and κ-light chain concentrations were 43.8 g/L (normal range, 8.7–17 g/L), 2455 IU/mL (normal range, 10–340 IU/mL), and 515 mg/dL (normal range, 3.3–19.4 mg/dL), respectively. Serum protein electrophoresis disclosed a monoclonal spike in the γ-globulin region and urine electrophoresis also revealed a monoclonal spike. Serum immunofixation electrophoresis confirmed the presence of an IgG-κ chain monoclonal M component. A bone marrow (BM) examination showed that plasma cells and eosinophils were 16.2% and 28.6%, respectively (Fig. 2). A karyotype analysis showed 46,XX (20/20 cells). Interphase fluorescence chromosomal *in situ* hybridization (FISH) of BM cells revealed no gene abnormalities in 1q21, RB1, P53, D13S319, or IgH. A skeletal survey X-ray found no osteolytic lesions. A biopsy sample of accessory salivary glands showed no amyloidosis. A skin biopsy sample revealed LV showing angiocentric, neutrophilic segmental inflammation with endothelial cell swelling and fibrinoid necrosis on blood vessel walls (Fig. 3). A cellular infiltrate around the vessels showed leukocytoclasia of neutrophil nuclei. IgG or IgA deposits around the vessel walls were not clear. Although hypocomplementemia was noted, no manifestations suggesting autoimmune diseases and cryoglobulinemia were observed. Allergic purpura was less likely because of the absence of abdominal pain and arthralgia. Drug-induced purpura was also not suspected because no causative drug was being taken.

**Table 1:** Laboratory data before and after bortezomib, lenalidomide, and dexamethasone treatment

	(Normal range)	Before	After one course of VRD	After four courses of VRD	After eight courses of VRD
WBC (×10^9^/L)	(4–7)	23.3	5.4	6.1	4.3
neutrophil (× 10^9^/L)	(3.9–6)	9	4.1	3.4	2.1
eosinophil (×10^9^/L)	(0.2–0.4)	13.5	0.2	0.2	0.1
basophil (×10^9^/L)	(< 0.1)	0	0	0	0
lymphocyte (×10^9^/L)	(3.5–4)	0.5	1	1.7	1.6
monocyte (×10^9^/L)	(0.2–0.7)	0.3	0.1	0.8	0.6
RBC (×10^10^/L)	(380–500)	299	305	302	331
Hb (g/dl)	(12–16)	9.2	10.6	9.7	10.7
Plt (×10^9^/L)	(15–40)	152	286	195	197
Total protein (g/dL)	(6.9–8.2)	8.2	7.6	6.2	7
Albumin (g/dL)	(3.9–4.9)	2.16	3.3	3.8	3.9
LDH (IU/L)	(106–211)	280	204	140	160
AST (U/L)	(5–40)	50	15	10	15
ALT (U/L)	(5–35)	40	12	9	10
ALP (U/L)	(104–338)	1564	332	231	319
BUN (mg/dL)	(8–20)	47	17	23	20
Creatinine (mg/dL)	(0.4–0.8)	2.1	0.82	0.93	1.1
CRP (mg/dL)	(< 0.3)	6.6	0.13	0.03	0.04
CH50 (U/mL)	(31.6–57.6)	< 10	57.8	53.8	78.8
C3 (mg/dL)	(65–135)	17.2	81.4	91.9	113.3
C4 (mg/dL)	(13–35)	0	19	14.4	22.2
C1q (μg/ml)	(< 3)	8.2	< 1.5	< 1.5	< 1.5
Serum β2-microglobulin (μg/dl)	(< 2.0)	13.5	2.4	3.2	3
Serum immunoglobulin (Ig)					
IgG (g/dL)	(8.7–17)	43.8	13.2	11.5	1219
IgA (mg/dL)	(110–410)	96	114	112	184
IgM (mg/dL)	(35–220)	30	48	48	45
IgE (IU/mL)	(10–340)	2455	218	201	196
κ-light chain (mg/dL)	(3.3–19.4)	515	24.2	32.6	25.3
λ-light chain (mg/dL)	(5.7–26.3)	400	17.7	28	18.2
IFN-γ (IU/ml)	(< 0.1)	< 0.1	6.5	6.7	6.4
IL-4 (pg/mL)	(< 6)	50.3	< 6	< 6	< 6
IL-5 (pg/mL)	(< 3.9)	56.1	< 3.9	< 3.9	< 3.9
IL-6 (pg/mL)	(< 4)	76.2	3.4	< 4	< 4
IL-3 (pg/mL)	(< 31)	31	ND	ND	ND
IL-10 (pg/mL)	(< 5)	45	< 5	< 5	< 5
GM-CSF (pg/mL)	(< 8)	< 8	ND	ND	ND
TGF-β (ng/mL)	(1.56–0.24)	8.74	0.48	0.5	0.52

*ALP* alkaline phosphatase, *ALT* alanine transaminase, *AST* aspartate transaminase, *BUN* blood urea nitrogen, *CRP* C-reactive protein, *GM-CSF* granulocyte-macrophage colony-stimulating factor, *Hb* hemoglobin, *IFN-γ* interferon, *IgA* immunoglobulin A, *IgE* immunoglobulin E, *IgG* immunoglobulin G, *IgM* immunoglobulin M, *IL* interleukin, *LDH* lactate dehydrogenase, *ND* not done, *Plt* platelets, *RBC* red blood cells, *TGF-β* tumor growth factor-β, *VRD* bortezomib, lenalidomide, and dexamethasone, *WBC* white blood cells

The serum level of IFN-γ, which was secreted by Th1, was lower than 0.1 IU/ml (normal range, lower than 0.1 IU/mL) (Table 1). Serum levels of IL-4, IL-5, and IL-6 secreted by Th2 were 50.3 pg/mL (IL-4 normal range, lower than 6 pg/mL), 56.1 pg/mL (IL-5 normal range, lower than 3.9 pg/mL), and 76.2 pg/mL (IL-6 normal range, lower than 4 pg/mL), respectively. IL-3, granulocyte-macrophage colony-stimulating factor (GM-CSF), IL-10, and TGF-β levels were lower than 31 pg/mL (IL-3 normal range, lower than 31 pg/mL), lower than 8 pg/mL (GM-CSF normal range, lower than 8 pg/mL), 45 pg/mL (IL-10 normal range, lower than 5 pg/mL), and 8.74 ng/mL (TGF-β normal range, 1.56–0.24 ng/mL), respectively.

Our patient was diagnosed as having MM IgG-κ chain type, stage IIIA according to the Durie-Salmon system and stage II according to the International Staging System. Following a definite diagnosis, she received a VRD regimen: bortezomib, 1.0 mg/m^2^, days 1, 4, 8, and 11; lenalidomide, 15 mg/day, days 1–21, and dexamethasone 20 mg/day, days 1, 2, 4, 5, 8, 9, 11, and 12. After one course of the treatment, the cutaneous manifestation rapidly improved (Fig. 1b). Laboratory tests showed a WBC count of 5.4 × 10^9^/L, eosinophil cell count of 0.2 × 10^9^/L, RBC count of 305 × 10^10^/L, Hb concentration of 10.6 g/dL, and platelet count of 286 × 10^9^/L (Table 1). The serum total protein level was 7.6 g/dL, the LDH level was 204 U/L, the aspartate transaminase level was 15 U/L, the alanine transaminase level was 12 U/L, the alkaline phosphatase level was 332 U/L, and the C-reactive protein level was 0.13 mg/dL. The serum creatinine level was 0.82 mg/dL and serum levels of complement bodies were normalized. Serum concentrations of IgG, IgE, and the κ-light chain decreased (13.2 g/L, 218 IU/mL, and 24.2 mg/dL, respectively). The serum level of IFN-γ was elevated (6.5 IU/mL). Serum levels of IL-4, IL-5, IL-6, IL-10, and TGF-β decreased (< 6 pg/mL, < 3.9 pg/mL, 3.4 pg/mL, < 5 pg/mL, and 0.48 ng/mL, respectively). In the fluorescence-activated cell sorting (FACS) analysis of peripheral blood mononuclear cells, the ratio of Th1/Th2 increased. A BM examination showed decrease of plasma cells (3%). She achieved and maintained a very good partial response (VGPR) following VRD regimen for nine cycles without recurrence of LV and eosinophilia.

## Discussion

MM is a very heterogeneous disease comprising a number of genetic entities that differ from each other in their evolution, symptoms, response to therapy, and prognosis. The relationship between these symptoms and MM has occasionally been reported in the literature; however, the underlying mechanisms have not yet been elucidated in detail. The Th1/Th2 balance and Treg play an important role in MM [9, 10] and may be associated with these symptoms. We describe the first case of LV and eosinophilia in MM that was associated with a Th1/Th2 imbalance and Treg cells, and was successfully treated with VRD treatment.

The progression of MM has been attributed to different acquired changes in plasma cell behavior combined with evolving crosstalk between myeloma cells and different cell types within the BM microenvironment. Myeloma cells activate fibroblasts and endothelial cells, and stimulate immune and inflammatory cells. While the mechanisms by which neoplastic plasma cells affect the BM microenvironment currently remain unclear, in a previous study patients with early-stage and non-advanced MM were found to have a high Th1 proportion, whereas patients with advanced MM showed a predominant Th2-like response in peripheral mononuclear cells after stimulation by monoclonal IgG or microbial antigens (Ags) [9]. Therefore, microbial Ags-Th2 cells may be closely involved in the pathogenesis, recurrence, and development of MM.

LV is a systemic inflammatory disorder mostly involving small vessels and is characterized by: segmental, angiocentric, neutrophilic inflammation; endothelial cell damage; and fibrinoid necrosis [1]. In MM, LV is more frequently linked to cryoglobulinemia, infections, or medication hypersensitivity, and rarely occurs as a paraneoplastic syndrome. Moreover, LV has been reported to develop in progressive MM [2, 3]. The pathogenesis of LV may be attributed to the deposition of immune complexes that activate the classic and alternate complement pathways [11]. The production of C3a and C5a causes mast cell degranulation and neutrophil chemotaxis [12]. Neutrophils adhere to endothelial cells and migrate into the surrounding tissue in order to phagocytose and degrade immune complexes [13]. Damage to the vascular endothelium occurs via the lysosomal enzymes released during the course of neutrophil disintegration as well as the production of oxygen free radicals [14, 15]. The production of inflammatory mediators, including IL-6, increases the influx of neutrophils and induces the synthesis and expression of surface endothelial adhesion molecules [16]. IL-6 is a growth factor for myeloma cells that also promotes their survival and is produced in the BM microenvironment [6]. Increased levels of IL-6 are found in the serum of patients with MM, possibly as a result of its overproduction by BM stromal and bone cells [7]. The relationship between LV and MM may be attributed to high IL-6 levels, which contribute to the recruitment of neutrophils to the post-capillary venules of the dermis.

Eosinophilia, which may involve peripheral blood or tissues, may be associated with a wide variety of malignant tumors. Tumor-associated peripheral blood eosinophilia is often an indication of late-stage disease with widespread metastases, but is not necessarily an indication of late-stage disease and may correlate with a good prognosis in some types of malignant neoplasms [17]. Among hematopoietic tumors, Hodgkin disease and non-Hodgkin lymphoma of the T cell lineage are the most frequent, whereas MM is rarely associated with eosinophilia [18]. The major growth factors for eosinophils are IL-3, IL-5, and GM-CSF; however, IL-3 and GM-CSF were at normal levels in the present case. Serum IL-4, IL-5, and IL-6 levels increased in the present case when MM developed with eosinophilia, and these cytokines and eosinophils decreased after the treatment of MM.

The use of immunomodulatory drugs, such as thalidomide and lenalidomide, and the proteasome inhibitor bortezomib have been associated with improved survival in the relapsed and front-line settings, as recently reviewed. Bortezomib has been reported to affect myeloma cell growth by the blockade of NF-κB and down-regulation of cytokines, such as IL-6 [19]. Lenalidomide has been shown to reduce the number of Treg, activate CD8 T cells, and repair T-helper subsets with the Th1/Th2 response [20]. The VRD regimen has exhibited significant efficacy in the setting of newly diagnosed myeloma [21, 22]. In the present case, serum levels of Th1 cytokines (IFN-γ) were significantly reduced, while the levels of Th2 cytokines (IL-4, IL-5, and IL-6) and the Treg cytokine (IL-10 and TGF-β) were significantly increased; after two courses of the VRD treatment, IFN-γ levels increased, while those of IL-4, IL-5, IL-6, IL-10, and TGF-β decreased with improvements in LV and eosinophilia. The results of the present case suggest the reversal of the Th1 to Th2 shift through the inhibited secretion of TGF-β by MM cells and Treg cells using anti-myeloma therapy.

## Conclusions

To the best of our knowledge, this is the first case report of LV and eosinophilia associated with MM. Although the occurrence of LV or eosinophilia in MM is rare, these symptoms may be casually related to MM. An awareness of the possible paraneoplastic complications of MM is important for successful treatments with anti-myeloma therapy and the prolongation of survival. Moreover, this case suggests that the Th1/Th2 imbalance and Treg cells play an important role in progression and cancer immunity in MM. The present case reinforces the value of early evaluations for paraneoplastic symptoms in order to reach a diagnosis and allow for the prompt initiation of appropriate treatments and achieve successful therapeutic management.

## Authors’ information

SO and MN are treating patients of hematological disorders in Japanese Red Cross Society Wakayama Medical Center. KO is a pathologist in Japanese Red Cross Society Wakayama Medical Center.
